# Genomic and Postgenomic Approaches to Understand Environmental Microorganisms

**DOI:** 10.1155/2018/4915348

**Published:** 2018-10-24

**Authors:** María-Eugenia Guazzaroni, Raul Alberto Platero, Rafael Silva-Rocha

**Affiliations:** ^1^Faculty of Philosophy, Sciences and Letters at Ribeirao Preto, Ribeirao Preto, Brazil; ^2^Clemente Estable Biological Research Institute, Montevideo, Uruguay; ^3^Ribeirao Preto Medical School, Ribeirao Preto, Brazil

The introduction of new high-throughput technologies has profoundly impacted the development of modern science outside the gene-centered understanding perceived in the earlier genomic era [1, 2]. Thus, low-cost platforms known as Next Generation Sequencing (NGS) technologies can produce millions of sequences of DNA molecules with different yields and sequence lengths, having displayed a considerable reduction in cost along the last decade and a lustrum [3].

At the same time, microorganisms are the most abundant organisms on Earth, playing elemental roles in biogeochemical, biomedical, and biotechnological processes. In this sense, novel problem-solving techniques involving systems and synthetic biology approaches have emerged to give significant insights into the comprehension of the molecular mechanisms related to microbial function. Moreover, this knowledge can be extended to our understanding of microbial processes, allowing the exploration of environmental microorganisms—including bacteria and fungi—as cell factories. In this context, this special issue attempts to explore how postgenomic approaches have allowed to take advantage of the abundance of the genetic and biochemical information currently being produced from the fields of (meta)genomics, (meta)transcriptomics or transcriptome profiling, and (meta)proteomics and how large amounts of data generated by these methodologies are integrated to engineer microbes for relevant applications.

Accordingly, the current special issue includes two original researches and three review articles, comprising different aspects of the selected topics. For example, in one article, L. F. Ribeiro et al. review the current strategies applied to overcome the limitations of Cas9 protein (CRISPR-associated protein 9) to generate robust and efficient tools for customized DNA manipulation using protein engineering approaches. Application of revised techniques in diverse biological processes should permit the optimization of gene therapy, metabolic flux, and synthetic gene networks. In another revision, L. de Fátima Alves et al. focus on how metagenomics has contributed to gain scientific understanding in diverse areas of knowledge. In this framework, main milestones in the metagenomic field are presented over the last 30 years, since the first published metagenomic experiment. The article is oriented in a philosophical manner, providing perceptions into potentialities and current challenges of metagenomic approaches, presenting the field as a promoter of new concepts in microbial science. The revision article authored by R. G. de Paula et al. discusses new perspectives about control of secretion and cellulase expression based on RNA-seq and functional characterization data of the filamentous fungi *Trichoderma reesei*, one of the most well-studied cellulolytic microorganisms. In one research article, A. Sanches-Medeiros et al. report a new method to calibrate transcriptional activity using constitutive synthetic promoters in the bacterium *Escherichia coli*. In this article, authors demonstrate that simple experimental techniques involving the use of a single fluorescent reporter and plasmids are sufficient to provide robust characterization of transcriptional elements, which is fundamental to proper construction of complex synthetic circuits. Finally, in another research article, C. Mareque et al. evaluate how different levels of N fertilization affect the structure of total and diazotrophic endophytic bacterial microbiota associated with field-grown *Sorghum bicolor*. The data obtained by the authors contribute to the understanding of how agronomical practices impact the plant-associated microbiota, an essential step toward the design of new sustainable agricultural production systems based on plant growth-promoting bacteria.

Thus, in our opinion, gathered articles in this special issue would allow us to explore novel methodological approaches to understand the molecular mechanisms associated to microbial function. We hope that these materials will help to inspire readers to perform novel studies capitalizing the unlimited biological potential of environmental microorganisms, probably bringing important profits in areas of agriculture, industry, pharmacy, and biotechnology.
